# Supplementary material for: The interactome of intact mitochondria by cross-linking mass spectrometry provides evidence for coexisting respiratory supercomplexes
Source: Mol Cell Proteomics. 2017 Dec 8;17(2):216–32. doi: 10.1074/mcp.RA117.000470 (PMC5795388; doi:10.1074/mcp.RA117.000470)
Supplement: Supplemental Data [file supp_RA117.000470_133922_0_supp_23978_fzffwf.zip › spectra_annotation/mito_DR_spectra_annotation/413-1-35-1-13-1.pdf]

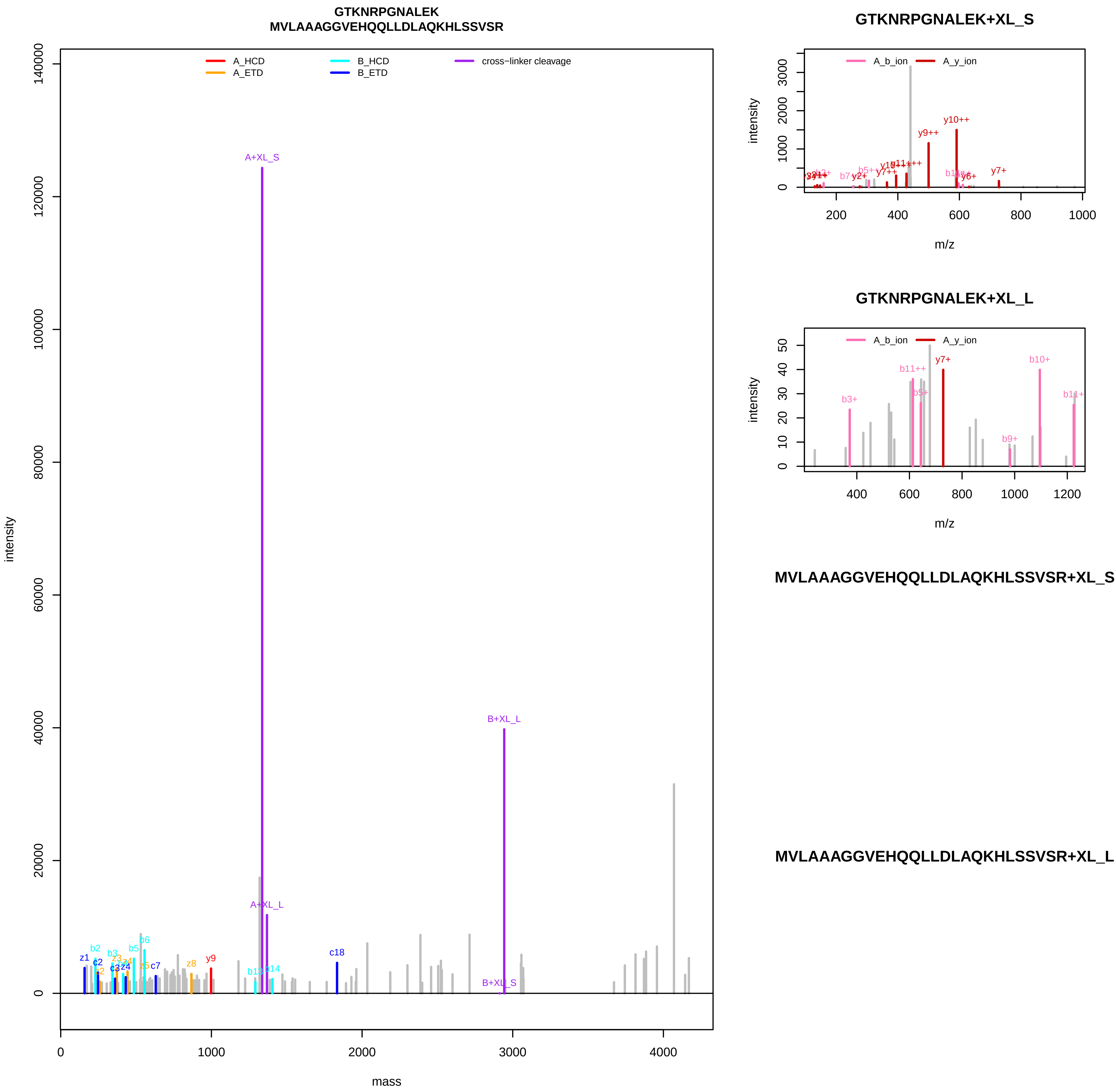
GTKNRPGNALEK
MVLAAAGGVEHQQLLDLAQKHLSSVSR
A_HCD
A_ETD
B_HCD
B_ETD
cross−linker cleavage
A+XL_S
B+XL_L
A+XL_L
B+XL_S
z1
b2
c2
b3
c3
z4
b5
b6
c7
z8
y9
c18
b14
intensity
mass
GTKNRPGNALEK+XL_S
A_b_ion
A_y_ion
y10++
y9++
y7+
m/z
GTKNRPGNALEK+XL_L
b11++
y7+
b10+
b3+
b9+
MVLAAAGGVEHQQLLDLAQKHLSSVSR+XL_S
MVLAAAGGVEHQQLLDLAQKHLSSVSR+XL_L
